# Osteogenic Sarcoma of the Maxilla: Neutron Therapy for Unresectable Disease

**DOI:** 10.1080/13577149977785

**Published:** 1999-09

**Authors:** Geoffrey L. Smoron, Arlene J. Lennox, James L. Mcgee

**Affiliations:** 1 Midwest Institute for Neutron Therapy at Fermilab P.O. Box 500 MS301 Batavia Illinois Provena Saint Joseph Hospital Elgin Illinois 60510 USA; 2 University of Illinois College of Medicine Peoria Illinois USA

## Abstract

*Purpose.* To present a case study involving the use of fast neutron therapy to treat an extensive unresectable osteogenic sarcoma arising from the left maxilla.

*Patient.* A 14-year-old male presented with a massive tumor producing severe distortion of his facial structures. He had already received six courses of chemotherapy, which had reduced his pain, but had not measurably reduced the tumor.

*Methods.* The patient was treated with 66 MeV fast neutrons to a dose of 20.4 Gy in 13 fractions over 35 days.

*Results.* CT assessments indicate gradually increasing calcification and noticeable reduction of soft-tissue disease in the frontal sinus, orbit and maxillary antrum.There has been some recontouring of the facial structures.The boy conducts an active life, has no pain, and feels well. He was 17 years old at the last follow-up.

*Discussion.* Fast neutrons have a greater biological effectiveness than conventional photon beams. Their use has been associated with improved chance for local control of unresectable disease.This case illustrates their effectiveness in controlling an unusual and aggressive osteogenic sarcoma of the facial bone and sinuses.


*Sarcoma* (1999) 3, 141–144

CASE REPORT

### Osteogenic sarcoma of the maxilla: neutron therapy for unresectable disease

GEOFFREY L. SMORON,[1] ARLENE J. LENNOX[1] & JAMES L. MCGEE[2]

[1]*Midwest Institute for Neutron Therapy at Fermilab, Batavia, Illinois Provena Saint Joseph Hospital, Elgin, Illinois, USA*
[2]*University of Illinois College of Medicine, Peoria, Illinois, USA*

**Abstract**
*Purpose.* To present a case study involving the use of fast neutron therapy to treat an extensive unresectable osteogenic sarcoma arising from the left maxilla.
*Patient.* A 14-year-old male presented with a massive tumor producing severe distortion of his facial structures. He had already received six courses of chemotherapy, which had reduced his pain, but had not measurably reduced the tumor.
*Methods.* The patient was treated with 66 MeV fast neutrons to a dose of 20.4 Gy in 13 fractions over 35 days.
*Results.* CT assessments indicate gradually increasing calcification and noticeable reduction of soft-tissue disease in the frontal sinus, orbit and maxillary antrum. There has been some recontouring of the facial structures. The boy conducts an active life, has no pain, and feels well. He was 17 years old at the last follow-up.
*Discussion.* Fast neutrons have a greater biological effectiveness than conventional photon beams. Their use has been associated with improved chance for local control of unresectable disease. This case illustrates their effectiveness in controlling an unusual and aggressive osteogenic sarcoma of the facial bone and sinuses.

### Introduction

Osteogenic sarcoma occurring primarily in the maxilla is an unusual disease, one for which satisfactory treatment results are difficult to obtain.[1] Multi-modality therapy is recommended for the treatment of these tumors, because surgery is limited for osteogenic sarcoma arising in the maxilla, paranasal sinuses and mandible.[2,3] In these sites, radiation therapy plays a more prominent role as part of the definitive management.[4] Neutron therapy, in particular, is associated with an improved chance for local control of unresectable tumors compared to similar treatment using conventional photon radiation therapy.[5] We report here a case of a locally advanced osteogenic sarcoma that has responded to chemotherapy and neutron beam therapy.

### Patient

A 14-year-old Haitian male orphan was brought to the United States to undergo surgery on a large facial tumor. The surgeons considered the tumor to be unresectable and surgery was limited to biopsy. Pathological diagnosis identified the tumor as an osteogenic sarcoma. The patient received six courses of chemotherapy, which included one course of ifosfamide with Mesna and adriamycin, four courses of high-dose methotrexate with leucovorin rescue, and one course of cisplatin and adriamycin by continuous infusion. Subsequent to the completion of chemotherapy, the patient was referred for consideration of neutron beam therapy, having experienced reduction of his pain but no measurable tumor reduction.

Physical examination demonstrated a large mass producing severe distortion of the facial structures, with orbital hypertelorism secondary to tumor replacement, obliteration of the left nasal cavity, near obliteration of the right nasal cavity, and flattening of the nose over the facial surface with extension into the contralateral maxillary sinus. The left eye showed significant proptosis, with the eye displaced superiorly and laterally (Fig. 1). CT demonstrated tumor extending from the bulging left maxillary sinus into the nasopharynx, sphenoid bone, hard palate and nasal passageways, with nasal bone destruction and invasion into the left orbit (Fig. 2). The tumor measured 10.5 × 9.0 cm in greatest diameter as measured on the CT scan.

### Methods

The patient went through a full course of neutron therapy, receiving a dose of 20.4 Gy delivered in 13

Correspondence to: Midwest Institute for Neutron Therapy at Fermilab, P.O. Box 500 MS301, Batavia, IL 60510, USA. Fax: +1 630 840 8766.

1357-714X print/1369-1643 online/99/020141-04 © 1999 Taylor & Francis Ltd

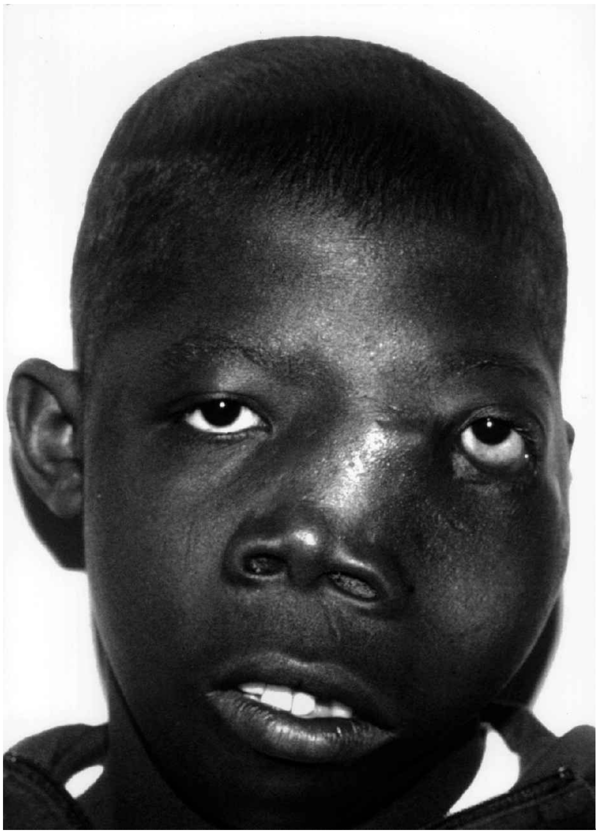

**Fig. 1.** *Pre-treatment facial hypertelorism caused by a large osteogenic sarcoma arising from the left maxilla..*

fractions over 35 elapsed days. The neutron beam is characterized as a p(66)Be(49) neutron beam produced by 66-MeV protons which strike a beryllium target to produce a neutron beam with depth dose characteristics similar to an 8-MV photon beam. The beam is fixed and the patient is treated isocentrically in the seated position utilizing a specially made chair which can be rotated. A computerized treatment plan required contouring in six planes to achieve an optimal dose distribution. Ten separate neutron beams were utilized to fulfill the treatment plan, which included four anterior beams, four left posterior oblique beams and two left anterior oblique beams. Wedges and multiple blocks in various beams were utilized as well. The patient completed the course of treatment nearly on schedule, although he required hyperalimentation and a short hospitalization during treatment.

**Results**

Follow-up examinations have demonstrated reduction in the tumor mass, gradually increasing calcification on CT scan, and noticeable reduction of soft tissue disease in the frontal sinus, orbit and maxillary sinus (Fig. 3). While there is persistent proptosis, the patient continues to have vision in the treated left eye. At last follow-up (December 1998), the 17-year-old boy conducts an active life, participates in sports, has no pain, and feels well. There has been

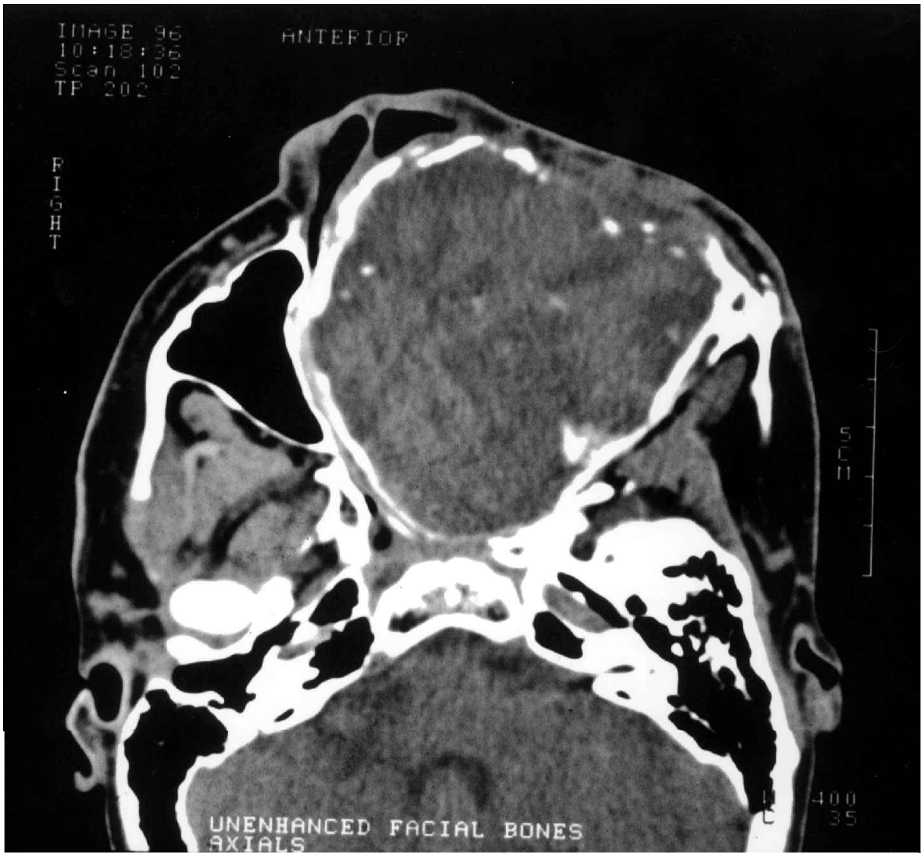

**Fig. 2.** *Pre-treatment CT scan showing a large mass in the left maxillary sinus, bulging into the right maxillary sinus, nearly obliterating nasal passages.*

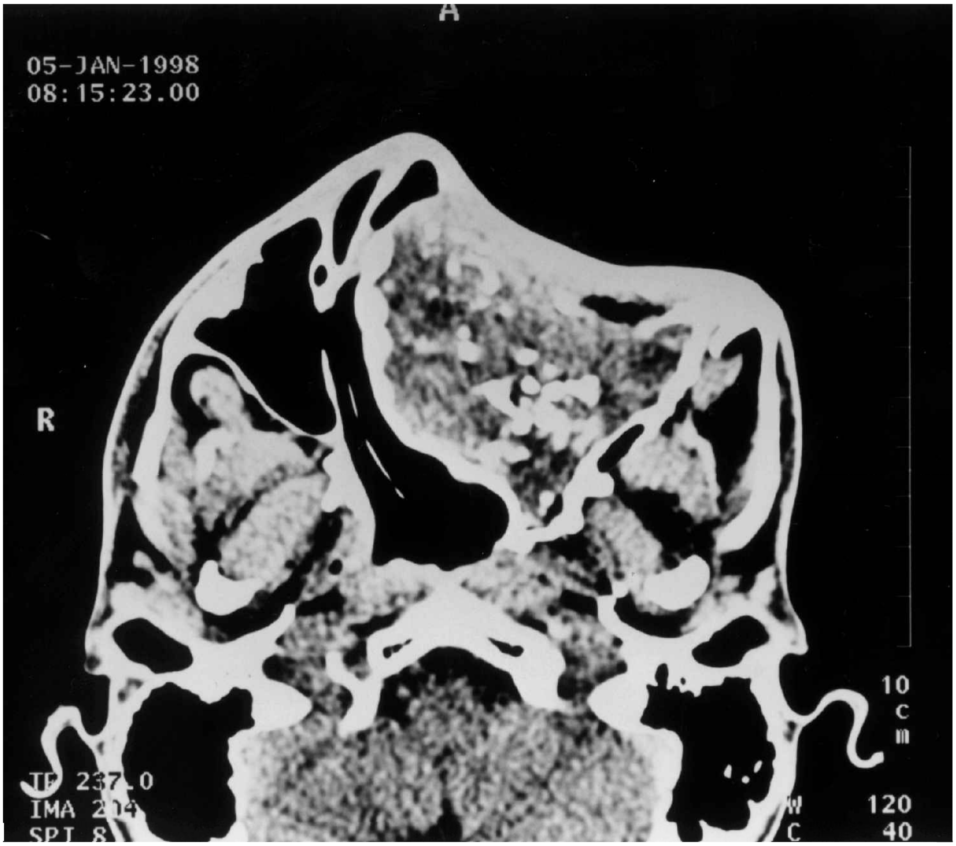

**Fig. 3.** *Post-treatment CT scan January 1998 demonstrating facial recontouring, reduction of tumor, and calcification within the tumor.*

some re-contouring of the facial structures associated with very slow regression of the mass (Fig. 4). On CT scan dated 12/98, the tumor mass measured 8.0 × 7.0 cm. Clinically there has been no evidence of local disease progression and no evidence of metastases.

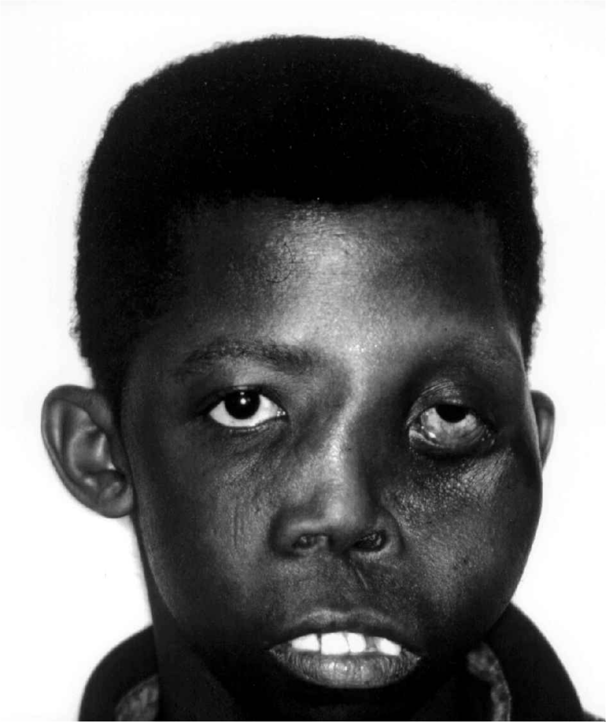

**Fig. 4.** *Post-treatment photograph demonstrating decreased hypertelorism and reduction of facial tumor.*

### Discussion

Osteogenic sarcoma of the facial bones and sinuses continues to be an unusual and aggressive disease, as 80–90% of all osteogenic sarcomas occur in the long bones.[6] Tumors of the maxilla account for only approximately 2–3% of patients.[1] This represents about one-third to one-half of osteogenic sarcomas classified as arising in the jaws, with mandibular lesions outnumbering maxillary lesions in most reviews.[7,8] For operable tumors, wide surgical resection is usually performed in conjunction with chemotherapy, and radiation therapy is often employed as well for close or positive margins, and inoperable and recurrent tumors. Recent clinical results have demonstrated an improving cure rate in the treatment of surgically resectable tumors when surgical margins are adequate.[9,10] However, the expectation for local eradication in surgically unresectable patients is poor, despite some historical results that demonstrate that tumors of the jaw tend to have a better prognosis than osteogenic sarcoma at other sites, with first evidence of failure more likely to be local recurrence than distant metastases.[9,11]

For this aggressive tumor, the radiobiological effect of neutron therapy compared to conventional photon therapy is greater, and may more likely result in local control of the tumor.[12] Historically, local control for unresectable and/or inoperable osteogenic sarcoma has been greater than 40% in patients (all age groups) treated at the Midwest Institute for Neutron Therapy at Fermilab.[13] Fast neutrons may be considered the best radiation quality used in the treatment of osteogenic sarcomas, as well as other sarcomas of soft tissue and bone in which the local control rate is 53%.[12] Worldwide data from earlier neutron therapy reports indicate a local control rate of 40–60% for sarcomas of bone and soft tissue, which compares favorably with the 21% local control rate reported utilizing photon irradiation.[5] For *high-energy* fast neutron beams, the likelihood and severity of soft tissue complications are similar to those of conventional photon therapy. In addition, there is approximately 25% less dose absorption in bony cavities due to the low neutron kerma in bone, thus lessening the risk of late injury to normal bone compared with conventional photon therapy.[14] Some early studies using *low-energy* fast neutron beams found unacceptable complications.[15]These low-energy beams are no longer recommended for treatment purposes, and all such facilities in the United States have been closed.

At present, there are only three high-energy fast neutron therapy facilities in the United States and a limited number of installations worldwide. Consequently, this treatment modality is of limited accessibility to patients, but nevertheless should not be overlooked as a possible way to improve control in appropriate cases.

### Acknowledgement

Work supported in part by the US Department of Energy under Contract No. DE-AC02-76CHO3000.

### References

1 Clarke JL, Unni KK, Dahlin DL, *et al.* Osteosarcoma of the jaw. *Cancer* 1983; 51:2311–6.
2 Marl RJ, Sercarz JA, Tran L, *et al.* Osteogenic sarcoma of the head and neck, the UCLA experience. *Arch Otolaryngol Head Neck Surg* (ALQ) 1991; 117(7):761–6.
3 Russ JE, Jesse RH. Management of osteosarcoma of the maxilla and mandible. *Am J Roentgenol* 1980; 140:572–5.
4 Dehner LP. Tumors of the mandible and maxilla in children. *Cancer* 1973; 32:112–20.
5 International Atomic energy Agency, Vienna, Austria. *Nuclear data for neutron therapy status and future needs.* 1997; IAEA-TECDOC-992:26.
6 Lewis M, Perl A, Som PM, *et al.* Osteogenic sarcoma of the jaw: a clinicopathologic review of 12 patients. *Arch Otolaryngol Head Neck Surg* 1997; 123(2): 169–74.
7 Batsakis JG. Osteogenic and chondrogenic sarcomas of the jaws. *Anal Otol Rhinol Laryngol* 1987; 96(4):474–5.
8 Garrington GE, Scofield HH, Bornyn J, *et al.* Osteosarcoma of the jaw: analysis of 56 cases. *Cancer* 1967; 20:377–91.
9 Van Es RJ, Keus RB, Vander Waal I, *et al.* Osteosarcoma of the jaw bone: long-term followup of 48 cases. *Int J Oral Maxillofac Surg* 1997; 26(3):191–7.
10 Cohen L, Hendrickson F, Mansell J, *et al.* Response of sarcomas of bone and of soft tissue to neutron beam therapy. *Int J Radiat Oncol Biol Phys* 1984; 10:821–4.
11 August M, Magennis P, Dewitt D. Osteogenic sarcoma of the jaws: factors influencing prognosis. *Int J Oral Maxillofac Surg* 1997; 26(3):198–204.
12 International Atomic Energy Agency, Vienna, Austria. *Nuclear data for neutron therapy: status and future needs.* 1997; IAEA-TECDOC-992:16-17.
13 Cohen L. Fermilab Neutron Therapy Facility experience 1976–1995. Paper presented at the *Neutron Therapy Symposium* in Cape Town, South Africa, 16 October 1995 (unpublished).
14 Bewley DK. *The Physics and Radiobiology of Fast Neutron Beams.* Bristol and New York: Adam Hilger, 1989; 27.
15 Glaholm J, Harmer C. Soft-tissue sarcoma: neutrons versus photons for post-operative irradiation. *Br J Radiol* 1988; 61:829–34.
